# Selections and Abstracts

**Published:** 1901-04

**Authors:** 


### SELECTIONS AND ABSTRACTS.

### INFANTILE COLIC AND COLIC IN INFANTS.

From time immemorial the aromatics or carminatives have been employed in the treatment of this affection, and whilst at the present day they still suffice for many, others have recourse to more potent drugs, the antispasmodics, and others again to still more powerful agents, as the opiates, chloral, hydrocyanic acid, etc., giving them either alone or in combination with some aromatic or antispasmodic. Even laxatives in very minute doses have found favor with some. The alkalies are sometimes prescribed with the view, it is said, of neutralizing a supposed unnatural acidity of the stools, but a scanning of the various formulæ will suffice to show that the small doses ordered cannot accomplish much, if anything, in that direction. I believe it to be more a traditional practice, which it is sought to place upon a rational basis.

The alcoholic stimulants, which in the very small doses are really aromatics, have long been, and still are, the remedy of the housewives and of the grandmothers.

The favorite prescription of one of the more ancient coryphées of medicine of this country, Dewees, was the following:

| | | |
|---|---|---|
| ℞ | Magnesia alba. usta | 1 scruple |
| | Tinct. fœtid.* | 60 drops |
| | Tinct. thebaic.† | 20 drops |
| | Aq. font | 1 ounce |

Of this twenty drops are given, and if the infant is not relieved in half an hour, then ten more are administered. "This dose is calculated for a child from two weeks to a month old. If it be older, a few more drops must be given ; and as the child advances in age or becomes accustomed to its use, the proportions of the ingredients must be a little increased."‡

* T nct. asafœtid.

† Tinct. opin.

‡ Dewees ; *loc. cit.* at end of article.

Starr, the eminent pediatrist of the City of Brotherly Love, makes a prescription as follows:

| | | |
|---|---|---|
| ℞ | Sod. bicarbonat | 16 grains |
| | Syrup | ½ fluidounce. |
| | Ag. meth. pip., enough to make | 2 fluidounces |

M. Sig.—One teaspoonful p.r.n. for a child of one month.

This prescription, he says, can be made more efficient by the addition of two drops of aromatic spirits of ammonia to each dose, or, in severe cases, one drop of spirits of chloroform.

He has found good results follow the administration of ten drops of gin in a teaspoonful of sweetened warm water.

Bromid of potassium and chloral he thinks most useful for the severe cases. This formula is the following :

| | | |
|---|---|---|
| ℞ | Potass. bromid | 16 grains |
| | Chloral hydrat | 8 grains |
| | Syrup | ½ fluidounce |
| | Ag. meth. pip., enough to make | 2 ounces |

M. Sig.—One teaspoonful for a dose. Can be repeated if necessary two to three times at intervals of half an hour.

John Thompson, of Edinburgh, believes that the best immediate treatment of an attack of colic consists of irrigating the lower bowel with a large quantity of warm water or administering a copious warm enema. The application of hot fomentations to the abdomen and of warmth to the feet are also serviceable, and twenty drops of whisky or a dose of carminative may help to relieve the child. An aperient is usually indicated to clear away irritating matter, and if the bowels are habitually constipated, this should be attended to. When there is obstinate recurring colic, small doses of codein ($\frac{1}{32}$ to $\frac{1}{24}$ of a grain) are occasionally useful as a temporary palliative while the diet is being gradually regulated.*

Vogel teaches that the treatment of an attack of colic consists in the cautious employment of narcotics, particularly the preparations of opium, of hydrocyanic acid and nux vomica, or in ethereal, aromatic remedies, chamomile, peppermint or melissa teas applied per os or per anum.†

* Clinical Treatment of Children, *Philadelphia Medical Journal*, October 28, 1899.

† Melissa calamintha smells like wild mint. It is used popularly as a tea in dyspepsia, in flatulent colic.

As already said, the home treatment of the mothers and grandames consists in the administration of some alcoholic liquor, as whisky or brandy or gin, or of a decoction of some aromatic herb, as peppermint, chamomile or catnip, or of seeds as fennel—the tea from the latter being particularly favored by German mothers.

In the earlier days of my practice, when crying infants were a sore trial, I also prescribed the opiates, making use of the various preparations, now of the camphorated tincture, paregoric, then of a certain supposedly denarcotized preparation of opium * and again of the simple tincture, usually ordering them in combination with aromatic syrups of rhubarb or syrup of ginger and some aromatic water.

Infants become readily accustomed to opium and some require larger and larger doses of it, and, therefore, in protracted cases, I preferred to avail myself of other medicaments. I now remember very well a most intractable case of infantile colic, in which all home remedies had been tried without benefit, for which I prescribed three drops of laudanum with five drops of whisky, to be given in two doses. At first, the one dose sufficed, then after a few days it required both doses to quiet the infant, and still a few days later even the two did not suffice and my juvenile friend kept up his shrieks as before. He would not be laid down on his bed at all, and the only way a moment's peace was gained was by holding him in arms and walking the floor with him.

Being afraid to increase the dose of opium beyond what had been already reached, I resorted to chloral hydrate and gave a prescription which I continued to use subsequently, and which I have always found effective where a soothing or hypnotic effect was desired. It reads thus:

| | | |
|---|---|---|
| ℞ | Chloral hydrate.......................... | 6 to 10 grains |
| | Mucil. G. acac.......................... | 1 dram |
| | Lac. asafetid .......................... | 2 drams |
| | Essent. anisi .......................... | ½ dram |
| | Aq. fenicul.......................... | 3 drams |
| | Sirup rhei. aromat.......................... | 1½ drams. |

M. ft. mixture. Sig.—One teaspoonful for a dose. To be repeated in 30 minutes, if necessary. For an infant from two to six weeks old. For older infants, the dose of chloral can be increased (to about 1½ grains).

* Which is said to be only about one third the strength of the simple tincture and from which the convulsivant elements, it is claimed, have been removed.

With larger experience I found that a more systematic treatment was required for a condition produced by dissimilar causes, than the mere routine prescribing the same formula in every case.

I also found that certain simple remedies acted as efficiently and as quickly, yes, even more so than the more powerful narcotics and hypnotics, and that, therefore, these could be dispensed with in most instances, a matter of certainly much moment to the infant, and of great advantage to it, as I consider.

In infantile colic, I have found nothing superior to the milk of asafetida. Made from the fresh gum, as directed in my book on Constipation, it brings relief in a very brief period after its administration. It is given, and in a few minutes thereafter, there begins a cannonading on the part of the infant, a discharge of flatus, that would do credit to a diet of beans; or eructations ensue. The cries cease, the frowns pass, and the child is again quiet, serene, smiles.

I direct that one third to one half teaspoonful be given, followed, if necessary (but this very rarely happens), in fifteen to twenty minutes by a second dose, to the youngest infant. A little fine sugar is placed upon the tip of the spoon (if preferred a drop or two of the syr. rhei. aromat. can be put there) and the spoon then placed to the infant's lips and the medicine allowed to flow in.

Infants, ninety-five out of every one hundred, take it readily, even like it, and there is, therefore, no struggling.

Occasionally, I make use of the following formula:

| | | |
|---|---|---|
| ℞ Lac. asafetid | ½ | ounce |
| Sirup manna | 2½ | drams |
| Essent. anisi | ½ | dram |
| Sirup rhei. aromat | 1 | dram |

M. Sig.—One teaspoonful for a dose.

The remedy is innocuous, and can, therefore, be placed with perfect safety in the hands of the mother or nurse, and its administration fully intrusted to them—a matter certainly of much moment for its future welfare, to the infant thus afflicted, often requiring, especially at the outset, two or three more doses per day, and that for a long period perhaps, of a medicine that shall be effective.

To show more clearly that I have not exaggerated as to the potent influence of asafetida on flatulent and spasmodic states of the intestine, the following case of an adult, in whom its use was attended with brilliant results, is here appended:

K. F., living in a large Western city, clerk, aged twenty-two. An inveterate cigarette smoker, a great drinker of coffee and most liberal consumer of whiskey. Since a number of years he had suffered from frequently recurring, violent attacks of pain in his bowels, which in the last year and a half have become so severe that hypodermic injections of morphia were often necessary to allay them. He was treated for dyspepsia, for liver trouble, and finally a condition of chronic inflammation of the intestine or a chronic appendicitis were suspected. He was badly run down, very much emaciated, and not able to do much. As he was in such bad shape and nothing seemed to benefit him at home, he was sent here to me. I examined him carefully, and found absolutely no pathological lesion of the intestinal tract. The other organs also appeared normal. I concluded that his pains were due to attacks of colic. I stopped his cigarettes, his coffee, his alcoholic drinks, and put him on a diet. The fourth day after his arrival here, and whilst in my office, he had a most frightful seizure. He became pallid, hands and face cold, features pinched, pulse small; he couldn't speak; it looked like approaching dissolution. I laid him on the sofa and then sought for some remedies. I happened to have a few ounces of milk of asafetida in the office, and this being first at hand, I poured out a full ounce, added twenty drops of Hoffman's anodyne and made him drink it. In five minutes thereafter he was up and declared he was feeling all right again; and in a further five minutes he left. He had no further attacks during the whole period of his stay here, and grew fat and stout. Returned home and again among his old compainions he resumed his old habits, and after a time the seizures again appeared, but never as violently as before, since he always cut them short and quickly relieved himself with a liberal dose of the asafetida, which he has put up for himself by the quart, and which he is never without.

There can be no possible objection to the remedy, except, perhaps, its odor; but I have never yet had a sensible mother object to its use on that account, when its more than overbalancing advantages were fully set before her.

When the infant is inclined to spit much after its administration, indicating evidently that it does not relish the taste, a few teaspoonfuls of sweetened warm fennel tea will at once wash away all the residue that may have remained in the mouth, both of medicine and odor.

I was informed by some very observant mothers, that a few teaspoonfuls of warm fennel tea increased the rapidity of action of the asafetida, and that with both the infant was relieved in a marvelously brief period.

When the attack comes on shortly after nursing (and this may occur even though we can detect nothing abnormal in the milk) we can frequently prevent it by giving the infant shortly before or very soon after nursing, a dose of the medicine. In older infants, four or five weeks, we can sometimes obtain the same result by preceding the nursing fifteen or twenty minutes with an ounce of warm, sweetened fennel ten, administered by means of the nursing bottle.

In very mild seizures, twinges only as it were, sweetened warm fennel tea, a few teaspoonfuls for the very young infants, an ounce or two, according to age, for the older ones, may quickly arrest the griping and thus give relief.

If the infant be inclined to be costive, an enema of warm water with a little sweet oil added thereto, or a soap suppository, may be directed as occasion may require, in addition to the medicine to be given internally.

In the colic due to overfeeding, to indigestion, to constipation, to improper food, I have found that the suffering was quickly relieved by a mixture made as follows:

| | | |
|---|---|---|
| ℞ | Mixt. rhei. et sod. | ½ ounce. |
| | Hoffman's anodyne | 40 drops. |
| | Syr. rhei. aromat. enough to make | 1 ounce. |

M. Sig.—Dose, ½ teaspoonful repeated in ½ to ¾ of an hour for an infant 3 to 4 weeks old. For younger infants the dose is 15 to 20 drops; for older ones from ½ to 1 teaspoonful, according to age.

Usually two, at most three, doses are all that are required.

If there be much overloading of the bowels, an enema, as described, or a soap suppository, in addition to the above, will be of much benefit.

If the colic be due to refrigeration, we will direct the application to the abdomen of dry heat, as a couple of layers of flannel, well warmed, or a tin plate heated and sufficiently covered with cloths, so as not to burn; we will have the feet wrapped in a warm cloth and we will order that the child be given a warm sweetened decoction of peppermint or fennel seed (infants generally seem to prefer the latter). By these measures we will quickly chase the furrows from the brow of our little patient and bring back his cherubim smile. He will once more be comfortable.

Sometimes the administration of a little good whisky, brandy, or gin—ten, fifteen, twenty drops (according to age) in a little sweetened warm water—will accomplish the same purpose and will bring quick relief.

Or the milk of asafetida with some aromatic, as in the formula above given, may be directed, and it will be found of great benefit. It is always to be preferred to the alcoholic stimulants with infants inclined to be nervous, or having a tendency to convulsive movements. Occasionally, in infants with a peculiarly sensitive intestinal tract, an opiate may be required, and here the camphorated tincture of opium, the paregoric of the Pharmacopœia, answers best, I believe.

℞ Tinct. opii. camphorat............... 1 fluidounce.

Aq. menth. pip....................... 5 to 5½ fluidounces.

Syr. rhei. aromat.................... 1 to 1½ fluidounces.

M. Sig.—Dose, ½ teaspoonful repeated in ½ hour if necessary. A further dose can be given in 2 hours.

It is only when this condition unquestionably exists that the administration of a preparation of opium in a case of simple colic (in an infant) is at all necessary or justified.—*H. Illoway in Philadelphia Medical Journal.*

---

**This Journal and Atlanta Semi-weekly Journal one year for $1.00 cash in advance. No checks. Strictly to new subscribers.**

[Selected.]

### CHARLATANISM IN THE CURE OF ALCOHOL, OPIUM AND OTHER DRUG INEBRIATES.

Within a few years the recognition of the curability of spirit and other drug habitués has brought into the field a large class of empirics who differ from the ordinary medical quacks in many respects. With but few exceptions, all these persons are habitués themselves or have been in some time past; hence they bring to the work a degree of sharpness and bold assumption which empirics in other fields lack. In many respects these quacks are better judges and have a clearer comprehension of the weakness and wants of their victims than others, but they lack in consistency and persistency. They are all of the dramatic type who pose for sudden, tremendous effect, and after a short, brilliant career disappear. Very few of them are before the world more than five or ten years. Unlike the patent medicine men, who year after year pursue a fixed course, these new charlatans have the gambler's spirit of quick returns for their labor and money. It may be said as a rule that they are more unscrupulous and more dishonest in their methods than other empirics, but fortunately in their zeal they exhibit this peculiarity, which often prevents the returns they expect. In my long experience as editor of a journal devoted to this specialty and as superintendent of an asylum for the treatment of inebriates, I have come in contact with many of these persons and have become familiar with their career. The "gold cure" delusion brought into activity the boldest and sharpest of this class. Expectation of large gains in a short time led to many very adroit schemes and plans which, if carried out, would have enriched their promoters to a fabulous extent. Many of these plans were submitted to me, with the drugs which they claimed to have discovered, and great efforts were made to enlist the Journal and my personal interest in their promotion. The assumption and arrogance of these "discoverers" was almost phenomenal. I was approached over and over again with offers of fabulous sums to engage in this or that enterprise. I found that all the leading spirits had been or were spirit- or drug-takers, and were buoyed up by delusional exaltations of gains, both pecuniary and physical. They were practically inebriates incapable of persevering effort, and unable to bear misfortunes or disappointments. One concern started with a million dollars pledged, and extravagant expectations on paper; disappointments of the first six months practically drove them out of business, yet their plans and management were of the very highest order. Another firm, officered by two inebriates who, in a state of great mental exaltation spent ten thousand dollars in circulars and paper plans, became discouraged and drank to death. Another syndicate went about purchasing property, making great show of permanency, and at one time had over three hundred different asylums in operation; then from some trivial causes broke up and abandoned them all. Most of these promotors had been patients formerly at different "gold-cure" homes, and believed they could do far more with their experience and knowledge of drugs than had been done before. Nearly all these institutions and projects were under the leadership of physicians who had been inebriates. Some of them were well educated and had been successful practitioners; others belonged to the irregulars whose early training was doubtful, and whose ethical sense was entirely wanting. They grouped certain combinations of drugs and claimed marvelous results from them, while in reality they were all of the same class and differed only in some minor particulars. The prescriptions used by these empirics are all combinations of strychnia, hydrastin, apomorphia, and cinchona barks. Some few mild vegetable narcotics which have been discarded by the profession were taken up again, but in all there were no remedies that were new to the profession, or had not been used before. The central object of the treatment was to destroy the alcoholic appetite, and this was claimed to be a cure. In many instances the surprise and joy in the patient's mind at the disappearance of all desire for spirits was accepted as evidence of final cure. This was followed by a species of hypnotism which lasted for a variable time and was in many cases very powerful, but when separated from the mystery and the surrounding psychical influence they reacted into the opposite condition. It was very soon evident that these empiric efforts were followed by transient results; then relapse and a worse condition than before. Still the impression remained on the minds of many persons that overcoming the drink taste was a real advance and gain to medical science. A number of physicians who claim respectability still persist in asserting that they have discovered a specific remedy for the cure of inebriety, but none of them will submit his prescription to a practical test. I have repeatedly offered to make such a test in my work and report the results above all possible collusion or bias, only asking that the drug be known and the matter be done openly and along scientific lines. This is refused for the most trivial reasons, and whenever the prescription has been shown it has been found to contain the common, usual drugs of that class. I have been puzzled to explain the fact that many of these physicians who advertise cures for the drink and drug states are credited with having been graduates of good colleges and been recognized as reputable men. In most instances I have found that they were drug-takers either openly or secretly, or had become disaffected and ostracized from the profession. In a few cases nothing but avarice of a low, grasping type seemed present. It is difficult to believe that these promotors of secret cures are honest and simply deluded, and yet, no doubt, instances of this kind can be found. One of the advertisers of secret opium specifics poses as a very reputable man in his community, and seems to have become a pessimist in regard to all professional honesty, and confirms it in his own conduct. This man charges fifty dollars in advance for treatment by his specific drugs, and advises the victim to pray with every dose taken, so as to secure a spiritual blessing. This counsel is very congenial to a large number of persons who entertain a high opinion of the physician, who combines religion and practice in this way. Another one of these charlatans sends a written guarantee agreeing to forfeit a thousand dollars if the patient is not cured, provided he follows the directions carefully. The medicines cost fifteen dollars a month. A bottle of this prescription contained aloes and morphia, with some other drug. The most prosperous of these quacks are those who send out drugs to be given in the tea and coffee secretly to destroy the appetite. This has become an enormous traffic in some sections, and many of these quacks have made large amounts of money. Recently traveling physicians have appeared, who for a certain stated sum promise a cure, giving their individual attention to each case. When the appetite is broken up the patient is called cured and the physician goes on. Another method is very common in all sections of the country. In this a genteel drummer calls on the family of the victim or some of his rich relatives, and advises some special course of treatment at a particular place. He puts himself forward as an example of the success of this method and solicits the influence and sympathy of the victim. In one instance a man of wealth whose nephew was an inebriate told me that he had six different drummers call on him within a few months to suggest the proper method of care and cure of his nephew. All these drummers claim to be actuated by philanthropy and desire to help others, for the benefit they have received. Another class of empirics have recently appeared who write that they have many inebriate patients, and will send them to this or that institution for a percentage or a fixed sum. This species of quackery has been manifest in many circles and among reputable men. Lower down but in the same class are the physicians who have made discoveries of wonderful prescriptions which they will sell to only a few persons. These prescriptions are warranted to cure inebrity. Some of the more thrifty of these empirics collect the names and addresses of inebriates and opium-takers, and also of their friends, with ratings of ability to pay, and offer these for sale. There are several lists in the market numbering many thousands of persons of this class who may be circularized and persuaded to take this or that method of cure. While it is very rare for any one who has been an inmate of an insane asylum to organize and conduct a similar institution, it appears to be the common ambition of reformed inebriates to engage in a work of curing others and to procure some specific drug for that purpose. No one seems to think that they are most *unfitted* for this work by their personal experience.

While the homes and sanitariums which have been opened for the treatment of spirit and drug habitués by specific methods have decreased rapidly within the last few years, their number is still large, and their work a menace to all scientific study. Such institutions have no regular organization, follow quack methods of attracting and holding patients, and are usually managed by unknown and so-called reformed men. Their remedies are always concealed, and they depend entirely upon boasted claims of remarkable cures.

These sanitariums assume exemption from all supervision and control by authorities on the ground that they only receive spirit- and drug-takers, and that these are moral disorders, not recognized as insanity; hence any one may open any kind of an asylum at any time and place and receive inebriates of all degrees, delirious, delusional, imbecile and insane, and treat them in any way possible, by any means, and turn them out at the will of the management. Everything depends upon the credulity of the patient and his friends and the mystery and pretension of the manager. This is a great wrong which should be corrected. All institutions of this class should be licensed the same as insane asylums, and come under some public supervision. This will be demanded in the near future. At present the army of drug-takers are by these quack methods and means made more incurable, and reputable asylums suffer with the victims in the value of the permanency of the work. Inebriety and other drug disorders are not cured by specific remedies or means of any kind, or secret remedies. No veritable remedy that will produce the results claimed for it needs to be advertised. Its merits will win beyond any advertising page. I have repeatedly offered to indorse and put before the public any remedy that could be found approaching a specific; but so far nothing has been offered. No advertised drug or method of cure or institution which promises to make a complete cure in a limited time is worthy of any credence. However much persons may be mistaken as to the effect of drugs, there have so far been no discoveries which will warrant the assertion that the degeneration from the drink craze can be restored in a few weeks or months.

From another point of view it is evident that the prevalence of quack methods and drugs is only the empiric stage of a great scientific advance in this field. This stage will give way to rational scientific measures and the present confused methods will be regarded with wonder. The prevalence of these quack methods and cures indicate the existence of a vast army of neurotics who are suffering from drug addictions. Literature has recently shown the widely extended influence of alcohol and opium in the production of serious disease. Notwithstanding all that has been said, there is little or no literature available to the student or physician, and the subject is practically ignored in all medical colleges. The recent graduate who is called to treat these cases has no practical knowledge or idea of the nature and extent of the damage from alcohol. The older practitioners are imbued with the moral theories of causation, and hence pay no attention to anything but the temporary relief of present conditions. No wonder the quacks abound and flourish in every community. Inebriety and other drug diseases should be recognized and taught as distinct neuroses occurring in every community and amendable to medical treatment. Then the reign of the quack and his methods will disappear.

T. D. CROTHERS, M.D.,
Supt. Walnut Lodge Hospital, Editor *"Journal of Inebriety."*
Hartford, Conn. —*Public Health Journal.*

---

### SOME MISTAKEN IMPRESSIONS REGARDING TROUBLES OF THE GENITO-URINARY ORGANS.*

It is only during recent years that much attention has been given to inflammatory diseases of the genito-urinary organs, and especially when the same has been caused by venereal infection.

For this reason many medical men, I might say the great majority, are working on theories at least one hundred years old and these having been carried down by medical writers until say from five to eight years ago. It must be surmised from the above statement, that graduates previous to that time are now working under these old theories, unless they are giving or have given most careful consideration to diseases of these organs since their college lectures. Even books of to-day are slightly hazy on certain conditions involving the genital and urinary organs. For instance, in the latest publication the opacity of urine in chronic urethral trouble is spoken of as being due to mucus and a slight amount of

* Read by J. Henry Dowd, M.D., Buffalo, N. Y., before the Physicians' Club, September 12, 1900, and at the thirty-third annual meeting of the Medical Association of Central New York, held at Rochester, N. Y., October 16, 1900.

pus, when in reality there is very little of the former, the turbidity in nearly if not every case being due to pus; the same being positively demonstrable if the microscope has been used. Many of these fallacious opinions have been so ground into the laity, that it is a common occurrence to hear a patient say that he had an inflammation of the bladder with his gonorrhea, where by careful questioning the fact is brought out that he was suffering only from an involvement of the posterior urethra. The diagnostician in every case was a doctor, one who has been taught that frequency of urination, with pus in the second glass, meant cystitis.

*Cystitis Complicating Gonorrhea.*—Although every case of acute gonorrhea in the male is accompanied by frequency of urination, still involvement of the bladder during this disease is of the rarest occurrence. To understand this thoroughly one should be intimately acquainted with the physiology of urination. In perfect health and under normal conditions, urine enters the bladder at the average rate of about two ounces every hour. When the viscus becomes filled to an extent sufficient to overcome the resistance of the internal sphincter, this muscle relaxes, allowing the fluid to trickle into the posterior urethra, which at this time becomes a part of the urinary bladder, the neck. If there is inflammation in this portion of the canal and it is acute, as soon as the first drop of urine strikes the inflamed membrane, there is immediate desire to urinate, and but little, if any, pus will be found in the second glass, for the reason that time did not permit the pent-up product of inflammation to gravitate backward and mix with the urine in the bladder.

On the other hand, supposing the membrane has been in a state of inflammation sufficiently long to withstand a certain amount of irritation, as soon as the internal sphincter gives way the urine enters, but the desire to urinate does not take place; rather the pus and other débris, intermingled with the incoming fluid, are carried into the bladder by the constant agitation which is going on, and when the desire is felt, if the urine is caught in ten divided parts, each and every one will contain pus, yet no involvement of the bladder is present.

As there is no doubt that the posterior urethra is involved in every case of gonorrhea, it must be very evident to all that the germs find their way, sooner or later, into the bladder. Various theories have been advanced as to the cause of gonorrheal cystitis, but I shall only offer an explanation as to why this viscus is so rarely affected. Although the epithelium lining the bladder appears similar to that of other parts of the anatomy, it seems to have a resisting power that is found in only one other structure, the vagina. Above the bladder is found two organs of the utmost importance to life, and it seems that nature has taken into consideration the frequency of urethral inflammation and has placed this barrier, the bladder, between this canal and the aforementioned organs. The female bladder also withstands the attack of inflammation well, yet it is more prone to inflammation than that of the male. It can be briefly stated that cystitis is rarely an accompaniment of acute, or even chronic, gonorrhea, when there is no other complicating factor present.

*Vaginitis.*—In the female it is known that there exists a direct route from the outside world to the peritoneal cavity, and it seems that nature has interposed a fortress between for the protection of that delicate portion of her anatomy. We find this tube, the vagina, to be lined with epithelium, which seems to have a resisting power against germ invasion and at the same time, and in contradistinction to the bladder of the male, the habitat of germs which, of no serious consequence to their possessor, has the power of destroying any foreign germ that may seek an entrance.

It may be inferred that the question will be asked, "how can pelvic peritonitis be accounted for, or, if infection does take place at the cervix, do not the germs gravitate downward in the secretions and in this way infect the vagina?" Suffice it to say, the resisting epithelium, together with the bacteria inhabiting the part, offer an obstruction so great that even the gonococci, in their most virulent form, except in extreme conditions of general systemic debility, fail to find a foothold in this portion of the human anatomy. Excluding traumatism, in the adult inflammation of the vagina is very, very rare. From careful examinations the writer has seen but two cases in five years; both were due to gonorrhea and both were syphilitics.

*Albumin and casts as indicative of nephritis.*—The old idea that when urine is boiled, becomes turbid, and this turbidity is not cleared away by the addition of nitric acid, that albumin is present, and that this is an indication of nephritis, is an exploded theory. It is not an uncommon thing to find the urine loaded with albumin during an attack of urethral inflammation where the most careful examination shows positively that no kidney lesion exists. It should be remembered that whenever serum exudes from the blood-vessels, albumin will be found in the urine, and the leaking of this substance (serum) is quite frequently found during an attack of gonorrhea or other inflammation involving the urethra or the bladder. Wherever there is a great amount of pus, the finest tests will always show a slight amount of albumin, but the kidneys must not be accused of producing the same, unless these organs are positively known to be involved. Albumin, in discoverable quantities, is not a constant accompaniment of Bright's disease; on the other hand, it is seldom found in the chronic forms, such as the interstitial. The finding of casts has generally been thought to be a most positive indication of nephritis, but such is not the fact in all cases. It is the most common occurrence to find a hyaline cast, especially if the centrifugal machine is used, and even epithelial casts are occasionally seen. If one will take the trouble to examine his urine carefully after a meal, rich in substances known to cause kidney irritation, also the free use of alcohol, many casts will be seen, hyaline, epithelial, and even granular; still, the kidneys are only suffering from a circulatory change. Of course, it must be inferred that the epithelium on the casts is in a healthy condition, for if it shows degeneration, it is indicative of a lesion.

*Syphilis.*—Many important points regarding this disease are open to doubt in the minds of not only the profession, but the laity. Syphilis must be included under the curable maladies, excepting where it assumes a malignancy from the outset, or is allowed to reach the tertiary stage. No attempt must be made to destroy a syphilitic chancre, as it very rarely accomplishes anything; but, on the other hand, causes a destruction of tissue which leaves a permanent scar. Tertiary symptoms may develop at any time, even as early as the sixth week after infection. The hot springs of Arkansas have no curative powers, they are only of benefit in cases of great debility, where the water acts as a tonic to the system. A positive prognosis must never be given in case of chancroid; there may be mixed infection.

*Stricture.*—Twisting or screw-like formation of the urine as it merges from the meatus is in no way indicative of urethral stricture. Herpes, in at least 95 per cent. of all cases, are nature's warning that trouble exists in the canal, and this trouble is generally stricture. A sound will not diagnosticate a stricture and locate it, unless it is a very small one and a large instrument is used. Where chronic urethral inflammation complicates a stricture, never attempt dilatation until the urine, by its clearness, shows that the general inflammation has almost, if not entirely subsided. The urethra, when to be operated upon, or manipulated, needs the same antiseptic precautions that any other part of the anatomy would receive. Internal medication is of little or no value in acute, and except in a very limited number of cases, is of no value whatever in chronic inflammation of the urinary tract, especially below the kidneys.

*Syphilis vs. gonorrhea.*—The common opinions held by the laity, that gonorrhea is no worse than a bad cold, and syphilis is never curable, and is transmitted to their children's children, is fast losing ground, and well it should. The statement can be made without much fear of contradiction, that of the two diseases syphilis is much the preferable, if we must choose between them. It is rare that death, or even much deformity, can be traced to syphilis, provided the disease is treated according to the methods in vogue to-day; whereas, on the other hand, no one can foretell the ultimate result which may follow an infection from the gonococci. It is a very broad statement, but could gonorrhea be eliminated from the category of diseases affecting humanity, fully 10 per cent. of the suffering that the human being is subjected to would be a thing of the past. It should be remembered that gonorrhea kills more people than syphilis, although the latter takes years of protracted treatment for a cure.—*Buffalo Medical Journal.*

### PECULIAR POPULAR REMEDIES OF THE BOERS.

A physician, writing in the Natal *Witness*, gives a very interesting and entertaining account of some of the domestic remedies in use among the Boers, with whose daily life, customs and habits he seems very familiar. He seems, however, to imagine that the strange medical superstitions given by him are peculiar to the Boers, which is far from being the case. In fact, there is not one of them, the belief in which is not shared by people of widely remote origin and separated from them by thousands of miles of space. Let us examine a few of them.

"Here," says the doctor, "is a remedy for toothache, which one of my patients explained to me, and when I expressed some doubt as to its efficacy, he offered to try it on me on the spot, but as I was not suffering and we could not find a suitable subject, it was unfortunately impossible to put it to the test. There is a small greenish yellow fruit, which is found in abundance on the veldt; the plant grows from half a foot high, and the fruit is about the size of an oak apple. Take several of these small apples, press the juice from them, and soak a piece of rag in it, then either wrap this rag round a candle or so smear it with tallow that it will burn. The afflicted person must hold his mouth open over the smouldering rag, so that the smoke enters; as this takes place small worms will be seen to fall out of his mouth, and thus, the cause of the pain being removed, your patient is cured. The remedy is said to be only of use when there are cavities in the teeth.

The belief that toothache is caused by worms hiding in the cavities of carious teeth, or burrowing in the gum, and that these worms may be smoked out by burning certain acrid drugs, is one common among savage and semi-civilized people, as among the Chinese, Tartars and certain tribes of American Indians and of Negroes. Among the Southern negroes a common remedy for toothache is dried chicken or pigeon dung, which is packed into the cavity of the aching tooth. Strange to say, it is generally efficient, a result probably due to the large proportion of uric acid present in the substance. The explanation given by the old darkies who most frequently administer the remedy is that it drives away the "weyums" (worms) from the root of the tooth.

"For the bite of a dog," says the correspondent, "the old saying is literally carried out: 'Take a hair of the dog that bites you'—for some of the dog's hair is taken and tied into the wound. Whether the dog's bite was dangerous or not, the remedy would probably produce a poisoned wound, giving great pain and in time discharging matter. As this developed, it would, no doubt, be regarded as evidence that the remedy was doing its work and removing from the blood the poison introduced by the dog. On this principle I have seen a small sore on the lip which a poor fellow had been persuaded was a cancer, turned into a large foul discharging swelling by the application of an irritating ointment, and the sufferer was told that this was the cancer being drawn out, and when I told him that the same ointment on the back of my hand (which was quite sound) would produce a similar sore, he did not seem pleased."

The very fact that it has grown to be a proverb, having an equivalent in nearly all languages, shows how universal is, or was, the belief in the efficacy of "the hair of the dog." As to the belief in the possibility of "drawing out" cancer and cancerous affections, it is that upon which ninety-nine out of every hundred of the so-called "cancer doctors" throughout America proceed to work their so-called "cures."

"The more sensational a remedy, the more acceptable it seems to be among the Boers. For inflammation of the lungs the warm skin, from a freshly-killed cat, is placed over the affected part, with the fur outwards. The great advantage of this remedy, is that if successful, it does not leave you in doubt, for if the inflammation is 'drawn out,' the fur falls from the skin. For the same disease there is another remedy, which, though somewhat disgusting, is very firmly believed in. A kind of tea is made, the principal ingredient of which is the dung of the goat, and this is given to the patient to drink."

The belief in the efficacy of the application of the skin of a freshly-killed fur-bearing animal in troubles of the chest arising from colds is another of those almost universal medical superstitions, but in this case, the method of ratiocination which gave rise to it is perfectly plain, which is the case in but few others. The belief in the falling of the fur, mentioned by the doctor, is as far as we can remember, peculiar to the Boers. It is, indeed, directly opposed to the principle underlying most of the superstitious practices in the treatment of disease—in fact, all of Shamanistic origin. These (the Shamanistic practices) are so devised that there shall be no direct evidence given to the patient, his relatives or friends, except such as the Shaman himself shall present, and declare to be favorable or the contrary.

As to the "disgusting remedy," the tea spoken of by the correspondent, as peculiar to Boerdom, it is, indeed, one of the most widely disseminated and most firmly believed in of the domestic remedies. Under the name of "sheep-saffron" or "goat saffron" it is, or was, a generation or so ago, an almost universally believed, in remedy among the negroes and ignorant whites of the Southern and Southwestern States of the Union. It is still in use in rural France, Germany and other Eurepean countries, as well as in South America. It is thought to be efficacious in intermittent and remittent fevers, biliousness, and of especial value in jaundice. This latter belief is, we think, a survival of the belief in the doctrine of signatures (*i. e.*, the idea that every medicinal substance bears in itself some physical quality which distinctly points to the diseases in which it will be found efficacious—thus, "sheep saffron tea" being of a deep greenish yellow hue, the hue of jaundice and of bile, is therefore efficient in diseases in which the bile is concerned).

As a remedy for inflammation, or to prevent it, our doctor says the Boers use strips of bacon bound to the afflicted part. This is another almost universal domestic remedy, and in certain conditions it seems to be based on the results of experience. A plough-boy in the Southern States who "snags his foot," the lad who steps on a nail or a piece of glass, the woodchopper who cuts a gash in his foot, leg or hand, in fact any one of the laboring classes who cuts or injures himself in any way, is not content until a strip of fat bacon is bound on the wound.

There are several other medical superstitions peculiar, according to the Natal physician, to the Boers, which we could show were simply shared by them with the ignorant and superstitious of other races, but we have already extended this notice beyond the limits at first intended.—*National Druggist.*

---

### LOCAL ANESTHESIA IN HEMORRHOIDAL OPERATIONS AND ALL VARIETIES OF MINOR SURGICAL WORK.

BY O. W. GREEN, M.D., Chicago, Ill., 1118 N. Y. Life Insurance Building.

Since there are so many people suffering more or less with hemorrhoids, and since orificial operations along that line have been performed only under general anesthesia, we desire to call attention to the fact that we have formulated a method by which hemorrhoidal operations are painlessly performed without the aid of general anesthesia. The operations are rendered painless by using the local anesthetic "Acestoria." This anesthetic is being used by hundreds of dentists throughout the country for the painless extraction of teeth.

Our method of operating on hemorrhoidal tumors is as follows: First, the patient is instructed to take a cathartic the night before the operation, and an enema in the morning. With a saturated solution of boracic acid thoroughly cleanse the rectum using syringe or otherwise, and then immediately inject every tumor in sight with "Acestoria," until each tumor is not sensitive to the prick of the needle. Sometimes it is best to use the bivalve speculum before, sometimes after injection, and sometimes not at all. It depends upon the condition and location of the piles. When it is more convenient to use the bivalve after injection, insert it and dilate the sphincter, and with hemorrhoidal forceps, or Pean's artery forceps, pick up the tumor at center, and turn it out. The bivalve is removed and inserted at right angles to first insertion, and another tumor is picked up and turned out as before, and so on until all are turned out.

We generally use the clamp method when possible. At this point we are ready to clamp, stitch and cut. Use Kelsey's or Pratt's clamp. After turning the tumors slightly outward with the forceps which were left hanging to them, each by turn is clamped at its base, being careful not to clamp either sphincter. Then with a straight needle put in two or more stitches, as may be needed back of clamp.

Now stitches are in place ready to tie as soon as clamp is removed and tumor cut off. Remove clamp and cut tumor with straight scissors through the white line made by the middle blade of the clamp. There will be no hemorrhage if this line is followed. The stitches are now tied. Each tumor is thus treated. Then with hydrozone and hot water, one part of the former to five of the latter, syringe or spray the field of operation thoroughly.

The object of using hydrozone is twofold: It is the safest and best germicide and hemostatic we have yet used, and we have tried many. Not being a poison, and depending upon the oxygen it contains for its action, renders it safe under all circumstances, both externally and internally.

As a dressing we have several times used nothing, simply cleansing with hot water and hydrozone. In other cases have used gauze, iodoform or carbolized, packing the rectum full, and leaving it there three or four days; and then either removing or giving cathartic and driving out the packing with the movement of the bowels.

An ideal dressing is ordinary sterilized gauze moistened with glycozone. Glycozone is anhydrous glycerine saturated with ozone, a powerful germicide and promoter of healthy granulation.

To prevent pain usually caused by the hypodermic needle, touch the point chosen for insertion with a glass-pointed rod, dipped in 95 per cent. carbolic acid.

In surgery of the nose, throat and ear, and also to avoid pain in local treatment, swab with cotton soaked with "Acestoria" every five minutes for fifteen minutes, or insert the pledget for ten minutes; but it is best to always use the hypodermic when possible.

To anesthetize the ear and stop earache, incline the patient's head to one side and drop into the ear about five drops, or sufficient to fill the external meatus.

Use "Acestoria" hypodermically in all cases where incisions or excisions are to be made, such as operations on ingrowing toe-nails, removal of splinters from the flesh, opening boils, abscesses, carbuncles, etc.

### THE SURGICAL DIAGNOSIS OF RIGHT-SIDE ABDOMINAL DISEASES.*

A group of recent cases of the right abdominal region calls attention to the interesting features of diagnosis of diseases common to that locality. This section presents more varied and common diseases than any other of the body. In its topography are included the right lobe of liver, the gall-bladder, duodenum, pancreas, hepatic flexures of the colon, right kidney and its suprarenal capsule, cecum and ascending colon, coils of small intestines, appendix and right ureter, and it is bounded posteriorly in part by the right psoas muscle, an important fact.

The most important organs, because of frequency of involvement in disease, are the appendix and gall-bladder in the male, tube and ovary in the female, while the kidney of this corresponding right side is very often surgically diseased. A specific and differential diagnosis is demanded, beginning with one of the more frequent diseased organs, the gall-bladder.

Acute inflammation of the gall-bladder manifests itself somewhat suddenly, with pain on right side of the abdomen, rapidly becoming general. A rapid and feeble pulse, quick thoracic breathing, fever, intense depression, marked tenderness on pressure, especially over the right side of the abdomen, rapidly developing tympanitis, persistent vomiting and an extremely anxious expression of countenance, are usually present. The acute peritonitis, localized at first, becomes general.

In all gall-bladder inflammations there is a tender spot, it may be called the "Mayo Robinson point," at the juncture of the outer one-third with the inner two-thirds of a line passing from the umbilicus to the ninth rib, at the notch found in the line of the costal margin.

For the appendix, let the same imaginary line pass from the umbilicus to the anterior superior spine of the ilium, and at the juncture of the outer third with the inner two-thirds of this line, will be found the "McBurney point." In the female it is somewhat lower.

* Read by A. L. Beahan, M.D., Canandaigua, N. Y., at the meeting of the Society of Physicians, Canandaigua, N. Y., February 7, 1901.

Neither of these points are the points of tenderness in cholecystitis and appendicitis exclusively, but they serve to fix the mind on two important points in a clear manner.

The right kidney projects one-half of its size below the costal border of the right lower rib. When the kidney is enlarged from disease or a perinephritic abscess, it projects forward as a more or less smooth mass, between a possible abscess of gall-bladder or appendix. It is never movable if a perinephritic abscess binds with adhesions posteriorly.

The right ovary and tube can be recognized by conjoined vaginal and abdominal palpation, when proper care is exercised. The diagnosis of disease of tube and ovary should be possible to every practitioner of medicine and surgery, as well as to the gynecologist, to the end that uterine tinkering in ovarian disease may not be practised, as any latent ovarian disease is easily excited or increased and the uterine mucosa is of itself but little influenced by ordinary local treatment, such as cleaning and applications.

Gall-bladder troubles of common character are from calculi in its cavity, its cystic duct, or in the common or hepatic ducts. If calculi are in either the hepatic or common duct, jaundice occurs; if in the cystic duct jaundice is not present. An enlarged gall-bladder may result from calculi, hydrops of the organ, empyema of the gall-bladder, or from a tumor essential to itself. A floating kidney often simulates gall-bladder disease, but the kidney can be pressed back into its original place, while the gall-bladder is less freely moved.

I have seen one gall-bladder filled with calculi that could be depressed below the umbilicus, making differential diagnosis uncertain, as jaundice had not occurred, because the calculi were of small size and could pass readily through the cystic and common ducts. But the appendix most taxes diagnostic skill. No symptoms are certain or peculiar to its manifestations of disease. No means of diagnosis are comfortably safe. The masters of the appendix art cannot impart exact rules. Leading symptoms are important, if present; equally important, if absent.

A recent case in a nurse we had watched a year, having a year previously treated her for rectal fissure. Only two symptoms at first were pointers—one, menstrual pains growing worse; another, constipation. You may say these are common to women. The patient at the menstrual epoch had a chill and belly pains, not so severe as to prevent caring for her patient night and day at the Natural Science Camp, Canandaigua Lake. After this no symptoms followed, except slight backache, malaise and vomiting, if cathartics were employed to relieve her constipation. The succeeding menstruation gave epigastric, followed by right inguinal, pain; then a cold spell, but not a distinct chill, followed by a temperature not above 101 1-5°, but never quite normal.

We often found 99 1-5° to be the appendical mean temperature. The pulse was never above 80. The menstrual pain, as presumed by patient, was a trifle more severe than that at the last time, and was ascribed to the fact that at the camp during the previous menstruation she might have taken cold. To increasing menstrual pains and constipation we could add but little history, not more than many women suffer, and if not in the hospital, I question if this attack would have been properly catalogued.

The bowels were freely moved, and palpation discovered a swollen and tender appendix. Operation was consented to and an appendix found swollen, with a phlegmon of meso-appendix of considerable size, so dark grey in color and so nearly boggy in feel, that the fear of its breaking down led to suturing it to the line of incision, so that if abscess developed it would be within easy reach; and for forty-eight hours our mind did not rest easy because of danger of sepsis. Two grape seeds were found in the appendix; one had much surrounding ulceration, feeding the phlegmon in meso-appendix with sepsis.

Two recent cases of appendicitis illustrate another puzzle in diagnosis. Both had attacks of peritonitis, one six weeks and the other twelve weeks previously. In either case the appendix was not to be palpated until the bowels were freely purged, and this was because in each case the appendix was posterior to the cecum, being sharply flexed on the cecum. The distention of the cecum prevented palpation of the appendix. The appendix may also be so badly disorganized as to be undiscoverable. The relation of menstrual irregularities and appendicitis seems to us to be intimate and to bear further research.

Usually the swollen appendix can be felt. With the patient in the dorsal position, legs well flexed, fingers of left hand applied to back of fingers of right hand, that the pressure may come from the left, and that with the right, so accustomed to sensitive touch, pressure may be made carefully and steadily against the psoas muscle during respiration. If the rectus comes up the hard pressure must be under and at the side of the muscle. A hard right rectus means appendicitis. If it continues after the patient is otherwise relaxed under an anesthetic, it means that pus is present. Painful micturition means that the appendix lies pointing toward the bladder. Pain in the back means a backward and upward pointing of appendix.

Most groin abscesses are from the appendix. Dr. Joseph Price recently asked the internes of several Philadelphia hospitals how many psoas abscesses they had seen in their terms of service. All answered none.

Perinephritic abscess pushes the kidney forward, and in a recent case the diagnosis was made correctly, as proven by operation. The kidney was well forward and, though much enlarged by the inflammatory process, continued to give its smooth, normal contour. The incision for abscess was made posteriorly. If made over prominence of swelling, to reach the abscess would require incision through the kidney, or a nephrectomy, a mistake recently observed, the kidney being needlessly sacrificed. In no other locality is a refinement in diagnostic acumen and ability more necessary. In no other does a similar gratification occur., The touch aids the judgment, the mind weighs evidence, and a correct diagnosis results.—*Buffalo Med. Journal.*

---

### EPIDEMICS OF SMALLPOX IN ANCIENT TIMES.

The ordinary reader who confines his readings to modern works, or the better known of the classics, even in medicine, can form no conception of the horrors of some ancient epidemics, or the ravages caused in former times by diseases now almost unknown or almost under the complete control of medicine. With the exception of the bubonic plague—the "Black Death" of the middle ages—probably the most dreaded and destructive of the old epidemic diseases was the smallpox, whose loathsome nature, combined contagiousness and infectiousness as well, and its intense malignity, increased by the utterly unreasonableness of the methods of treatment from time to time in vogue, all tended to make it the scourge of mankind. While to-day the spread of the disease is limited and confined to the more ignorant classes by the almost universality of vaccination, and its loathsome nature largely modified by the progress of hygienic science, in those times—or prior to the introduction, first of inoculation, and subsequently of vaccinatio—it attacked alike princes and peasants, kings and queens and royal families in their palaces, lords and ladies of high degree; the knight in armor and his squire; the citizen and his dame and family—all were subject alike with the humblest kern in his hut, or the beggar in rags on the highway, to be stricken down by the "Red Terror."

It swept away towns and made waste the country by the enormous mortality it occasioned. More than this, it terminated ancient dynasties and thus changed the fates of nations. Among the many such results we may point to the epidemic in Middle Gaul toward the end of the sixth century, as related by Gregory of Tours.

This epidemic broke out in the month of August, 580 A. D., appearing first in Central Gaul, but soon covering the whole region now known as France. Gregory of Tours speaks of the disease as a "*Morbus dysentericus--procedentibus erumpentibusque vesicis,decursa sanie multa liberabantur,*" which Augustus Thierry, in his "*Récits des Temps merovingiens,*" after a most careful study, pronounced the disease to have been malignant smallpox—which from contemporary descriptions it undoubtedly was. The mortality was frightful, and especially among children and young people generally.

After striking Paris, the disease penetrated Soissons and entered at once the royal palace of Braine, then occupied by Chilperic. Its first victim was the monarch's youngest son, Dagobert, "not yet baptized," we are told by the chronicler as a matter of highest importance, "but rallying somewhat, the rite was administered." The next, but almost simultaneously, was Chlodobert, next in age, but

15 years old. The queen, the celebrated Fredegonda, was next stricken. The children were treated with "herbs and potions," but the queen depended on religious services; all, however, died, and in the order named. Chlodobert, always sickly, was given a mixed treatment, as it might be called, "roots and herbs," but was also "*exposed near the tomb of St. Medard, in the basilicon of that saint, at Soissons, having been borne thither from Braine for that purpose.*" The poor child died, as we are informed, "on that day, even that same minute" (*i. e.*, minute of exposure).

What the political consequences of this epidemic were and their effect on the future of Gaul is a matter of history.—*National Druggist.*

---

### SEXUAL NEURASTHENIA.

BY D. J. McCARTHY, M.D.,

Associate in Medicine, Wm. Pepper Clinical Laboratory, University of Pennsylvania.

Sexual neurasthenia is a fatigue neurosis, either the result of sexual excess or sexual abuse, or a neurosis dependent on physical or psychical deterioration which is traced in the mind of the patient to some fancied or real perversion or abuse of the sexual function. A large percentage of the cases of sexual neurasthenia coming under the care of the nerve specialist are engrafted on a defective, unstable nervous system. It is, indeed, this defective psychic make-up of such individuals, this lack of mental equilibrium and inhibitory power which leads to the reckless abuse of the normal sexual function, or the more common perversion of it, excessive masturbation. It is therefore easy to understand how the continued drain on these generative organs leads to a depression in the general physical tone, and secondarily to the worry and introspection which is almost always associated with it.

A normal, healthy body will stand a great deal of sexual drain, provided it be unassociated with worry and mental strain; but just so soon as these elements are added, the loss of flesh and strength and tone rapidly takes place, and by counteracting on each other lead to the physical and mental wrecks we see in the terminal sexual neurasthenic. Given an individual addioted to any of these abuses of the organism, one can almost predict the course of the disease. In the normal sexual act, a lessening of the sexual appetite or vigor, or in the perverted forms an accidental discharge of seminal or prostatic fluid, or perhaps a sequence of nocturnal emissions, is the starting point of the introspection which persists and grows as long as the disease lasts. These accidents will naturally occur when the effects of the sexual drain have become manifest on the physical being; the excessive tissue waste having been thoroughly inaugurated before the mental strain has developed.

The average individual, if the will power be sufficiently strong, will now avoid his primary error, only to find that the over-active glands, in their efforts to get rid of a surplus secretion, have produced nocturnal emissions, involuntary seminal discharges, etc., which to him are retribution for his past sins, and add fuel to the smouldering fire which now bursts forth into flame. The mind, during every spare moment, is directed to the sexual apparatus, only to find a new sympton at each turn. His active life and exercise is dispensed with, that he may brood over his misfortune, and try all the patent nostrums for the return of his lost manhood. The muscles become flabby and weak. The liver, in its turn, fails to take care of the added work, and constipation, with anorexia and nervous dyspepsia, completes the wreck so easily begun. In the meantime he has exhausted all the patent nostrums for the relief of his disease, only to find that the temporary improvement due to the mental impression rapidly disappears, leaving him worse off than when be began.

The advertising doctor is next given a trial, with the same result, and finally, without mind or body or occupation or money, he turns to the nervous dispensary, to find many kindred spirits, when he thought he must be the only one so sorely afflicted.

The patient is usually depressed, with sallow, unhealthy skin, dilated pupils, flabby muscles, very restless and irritable; and with all the pains and aches and diseases he has ever heard detailed. Headache and backache and tremor are usually present. Lack of concentration of thought which the patient thinks is loss of memory and "giving way of the mind" is characteristic. Slight muscular effort produces fatigue, and an accentuation of the other symptoms. The reflexes are exaggerated, the urine is scanty and throws down a heavy precipitate of phosphate, and not infrequently contains spermatozoa and semen crystals. Constipation is almost invariably present, and a flabby, coated tongue and fetid breath are the external evidences of the perverted digestive functions. Indeed, there are but few, if any, of the bodily tissues performing a strictly normal function in the terminal cases of sexual neurasthenia. The clinical picture of the "essential neurasthenic," a neurasthenia due to mental strain, overwork, etc., etc., does not differ essentially from that given above. Neurasthenia, independent of its cause, follows a distinct clinical type, the course and prognosis depending, of course, on the ease with which the previous errors are abandoned and the disposition of the tissues to regain their normal function. Sexual neurasthenia is more prone, I think, to result in a complete loss of the mental equilibrium (sexual insanity) than the other forms of neurasthenia. This is especially true if the neurasthenic comes from an insane stock. The proportion of cases with this termination is very small indeed.

The treatment of sexual neurasthenia does not differ materially from that of the other forms of neurasthenia. A general elevation of the tone of the entire system, and with it of the exhausted nervous system, is necessary, and best attained by a thorough systematic course of rest treatment in a private hospital or sanitarium. The daily visit of the physician, with the encouragement and reinforcement of the increments of moral tone which comes with the correction of the vicious errors in metabolism, are as necessary as the treatment of the physical being. The bromids, hyoscin, etc., may at first be necessary to control the sexual hyperesthesia, but should be dispensed with as early as possible. Local treatment is usually unsatisfactory, as it tends to keep the patient's mind on the sexual organs, when it should be directed into more normal channels.—*International Medical Magazine.*—*Canadian Practitioner.*

